# Author Correction: Performance and usefulness of a novel automated immunoassay HISCL SARS-CoV-2 Antigen assay kit for the diagnosis of COVID-19

**DOI:** 10.1038/s41598-021-03518-y

**Published:** 2021-12-08

**Authors:** Kaori Saito, Tomohiko Ai, Akinori Kawai, Jun Matsui, Yoshiyuki Fukushima, Norihiro Kikukawa, Takuya Kyoutou, Masayoshi Chonan, Takeaki Kawakami, Yoshie Hosaka, Shigeki Misawa, Haruhi Takagi, Yasushi Matsushita, Makoto Hiki, Atsushi Okuzawa, Satoshi Hori, Toshio Naito, Takashi Miida, Kazuhisa Takahashi, Yoko Tabe

**Affiliations:** 1 Department of Clinical Laboratory Medicine, Juntendo University Graduate School of Medicine, 2-1-1, Hongo, Bunkyo-ku, Tokyo, 113-8421 Japan; 2 Clinical Innovation, Sysmex Corporation, Kobe, Japan; 3 LS Medical Affairs, Sysmex Corporation, Kobe, Japan; 4 Engineering 1, Sysmex Corporation, Kobe, Japan; 5 Department of Clinical Laboratory, Juntendo University Hospital, Tokyo, Japan; 6 Department of Respiratory Medicine, Juntendo University Graduate School of Medicine, Tokyo, Japan; 7 Department of Internal Medicine and Rheumatology, Juntendo University Graduate School of Medicine, Tokyo, Japan; 8 Emergency and Disaster Medicine, Juntendo University Faculty of Medicine, Tokyo, Japan; 9 Department of Cardiovascular Biology and Medicine, Juntendo University Faculty of Medicine, Tokyo, Japan; 10 Department of Coloproctological Surgery, Juntendo University Graduate School of Medicine, Tokyo, Japan; 11 Department of Infection Control Science, Juntendo University Graduate School of Medicine, Tokyo, Japan; 12 Department of General Medicine, Juntendo University Graduate School of Medicine, Tokyo, Japan; 13 Department of Next Generation Hematology Laboratory Medicine, Juntendo University Graduate School of Medicine, Tokyo, Japan

Correction to: *Scientific reports* 10.1038/s41598-021-02636-x, published online 01 December 2021

The original version of this Article contained an error in the Abstract.

“The best cut-off index was determined, and clinical performance was tested using 115 serum samples obtained from 46 patients with coronavirus disease 2019 (COVID-19) and 69 individuals who tested negative for COVID-19 through reverse transcription quantitative polymerase chain reaction (RT-qPCR).”

now reads:

“The best cut-off index was determined, and clinical performance was tested using 115 nasopharyngeal swab samples obtained from 46 patients with coronavirus disease 2019 (COVID-19) and 69 individuals who tested negative for COVID-19 through reverse transcription quantitative polymerase chain reaction (RT-qPCR).”

The original Article has been corrected

